# Treatment Regimens and Response Rates in Early TNBC: A Review of Real‐World Practice in the Second Decade of the 21st Century

**DOI:** 10.1155/tbj/9970072

**Published:** 2026-04-08

**Authors:** Maria Eleni Hatzipanagiotou, Verena Tannert, Michael Gerken, Miriam Pigerl, Sophie Räpple, Jonas Roth, Monika Klinkhammer-Schalke, Olaf Ortmann, Stephan Seitz

**Affiliations:** ^1^ Department of Gynecology and Obstetrics, Comprehensive Cancer Center (CCC), Würzburg, Erlangen, Regensburg, Augsburg (WERA), National Center for Tumor Diseases (NCT), University Medical Center Regensburg, Regensburg, Germany, ukr.de; ^2^ Bavarian Cancer Registry, Regional Center Regensburg, Bavarian Health and Food Safety Authority, Regensburg, Germany; ^3^ Tumor Center Regensburg - Center for Quality Management and Health Services Research, University of Regensburg, Regensburg, Germany, uni-regensburg.de

**Keywords:** clinical cancer registry data, neoadjuvant chemotherapy, real-world data, triple-negative breast cancer

## Abstract

**Background and Aims:**

Real‐world evidence on the treatment of early triple‐negative breast cancer (TNBC) remains limited. This study provides an overview of neoadjuvant chemotherapy (NACT) regimens used in the 2010s of the 21^st^ century, analyzing patient outcomes and treatment patterns based on real‐world data from a large population‐based cancer registry.

**Methods:**

In this retrospective, noninterventional, single‐center study, we analyzed data from TNBC patients diagnosed between January 1, 2010, and December 31, 2018, registered in the Tumor Centre Regensburg. Data included demographics, pathology, treatment regimen, recurrence, and survival, with follow‐up extending to December 2023. Outcomes included pathologic complete response (pCR), overall survival (OS), and recurrence‐free survival (RFS).

**Results:**

A total of 319 patients were included. Among them, 132 patients (41.4%) received NACT with epirubicin/cyclophosphamide (EC) and paclitaxel (EC‐T), 74 patients (23.2%) received NACT with EC‐T and platinum (EC‐P/T), and 22 patients (6.9%) received NACT with EC‐P/nab‐paclitaxel (EC‐P/nabP). Other NACT protocols were administered in 91 (28.5%) patients. A pCR occurred in 49.8% of NACT patients, with a 37.1% rate in the subgroup without platinum. Addition of platinum significantly increased the pCR rate to 54.1% compared to EC‐T (odds ratio [OR] 3.476, 95% confidence interval [CI] 1.655–7.300, *p* = 0.001). EC‐P/nabP led to a significant increase in pCR rate to 77.3% (OR 8.767, 95% CI 2.421–31.744, *p* < 0.001).

**Conclusion:**

The evidence from clinical trials was quickly incorporated into clinical practice, leading to higher pCR rates and benefits for patients. In the next step, the implementation of immune checkpoint inhibitors in real‐world practice will be analyzed.

## 1. Introduction

Approximately 15%–20% of invasive breast cancers lack hormone receptor expression and human epidermal growth factor receptor 2 (HER2) overexpression or amplification [1, 2], falling under the category of triple‐negative breast cancer (TNBC). These cases are associated with an increased recurrence risk and poorer survival [3]. Neoadjuvant chemotherapy (NACT) followed by surgery is the standard therapy for early‐stage TNBC, allowing treatment response evaluation and personalized postneoadjuvant strategies [4–8]. Attaining pathologic complete response (pCR) significantly improves outcomes, but conventional regimens yield pCR rates in only about one‐third of patients [9–11]. In the 2010s of the 21^st^ century, increasing evidence emerged supporting the integration of carboplatin into NACT protocols, significantly improving pCR rates in patients diagnosed with early‐stage or locally advanced TNBC. The rationale for this is that the cytotoxic platinum derivatives are DNA‐damaging agents that induce DNA strand breaks, consequently triggering cell apoptosis [12]. Based on this biological rationale, several studies have evaluated the impact of adding platinum‐based agents to standard chemotherapy. In two randomized Phase II trials, the inclusion of carboplatin led to noteworthy outcomes. The CALGB/Alliance 40,603 trial, which involved 443 patients with Stage II to III TNBC, demonstrated that adding carboplatin to weekly paclitaxel, followed by doxorubicin plus cyclophosphamide (with or without bevacizumab), increased the pCR rate from 41% to 54% [13]. Similarly, in the GeparSixto trial with 315 patients diagnosed with Stages II–III TNBC, integrating carboplatin into a regimen of weekly paclitaxel and liposomal doxorubicin, alongside bevacizumab, raised the pCR rate from 43% to 53% [14]. Consistent with these findings, in the BrighTNess Study, a Phase 3, randomized, double‐blind, placebo‐controlled trial involving 634 patients with clinical Stages II–III TNBC, a pCR rate of 58% could be achieved by adding carboplatin to a regimen of weekly paclitaxel, followed by doxorubicin and cyclophosphamide [15]. Based on results from these studies, it has become evident that the addition of carboplatin in the neoadjuvant setting for TNBC leads to higher pCR rates. Achieving a pCR is correlated with improved event‐free survival (EFS). This has been demonstrated both in the long‐term analysis of the Brightness Study [16] and in the largest randomized controlled Phase three trial to date, which examined the efficacy and toxicity of adding carboplatin to standard chemotherapy in the neoadjuvant setting for TNBC [17]. However, the benefit of overall survival (OS) is not conclusively clarified [5]. The toxicity of chemotherapy is higher due to the addition of carboplatin [17]. Due to the not conclusively clarified influence on OS, the recommendations regarding the addition of carboplatin in national and international guidelines remain a topic of discussion [18–20]. A further improvement in pCR rates was achieved by replacing paclitaxel with nanoparticle albumin‐bound (nab)paclitaxel, as investigated in the GeparSepto study [21]. Given the illustrated advancements in incorporating carboplatin and utilizing nab‐paclitaxel instead of paclitaxel, growing evidence has also been demonstrated in the field of immunotherapy for early‐stage TNBC: The data from KEYNOTE‐522 marked the beginning of a new era in the treatment of early‐stage TNBC. Since then, immune checkpoint inhibitors have firmly established their role in the treatment of both early and advanced TNBC. In the early 2020s of the 21st century, the combination of pembrolizumab with NACT, followed by postneoadjuvant pembrolizumab administration, has become the established standard of care for Stages II–III TNBC [22]. In this retrospective review, we examine the evolution of treatment regimens for early‐stage TNBC in real‐world practice prior to the introduction of pembrolizumab. The objective of this study was to examine the integration of clinical trial evidence—specifically the introduction of platinum and nabpaclitaxel—into routine clinical practice and to evaluate their influence on pCR rates and survival outcomes. Utilizing population‐based data from the Tumor Center Regensburg registry, we systematically analyzed treatment regimens, therapeutic responses, and survival patterns to derive insights that may inform the optimization of future treatment strategies.

## 2. Materials and Methods

*Registry data source and coding standards* In this retrospective cohort study, clinical cancer registry data from the Tumor Centre Regensburg from patients with TNBC with a focus on diagnosis, therapy, and short‐ and long‐term outcomes were used for evaluation. A population of more than 2.2 million people, including Upper Palatinate and Lower Bavaria, is covered in this population‐based regional cancer registry. Electronic sheets of documentation contain information about diagnosis, course of disease, therapies, and the complete follow‐up of patients. These population‐based data originate from medical reports, pathology reports, and follow‐up records. Data collection is performed under state quality control and linked to vital status updates through regional and national health authorities. The Tumor Center Regensburg has been documenting tumor diseases in the Upper Palatinate and Lower Bavaria since 1991 and is integrated into the Center for Quality Assurance and Health Services Research at the University of Regensburg.

### 2.1. Patient Eligibility and Baseline Characteristics

**FIGURE 1:**
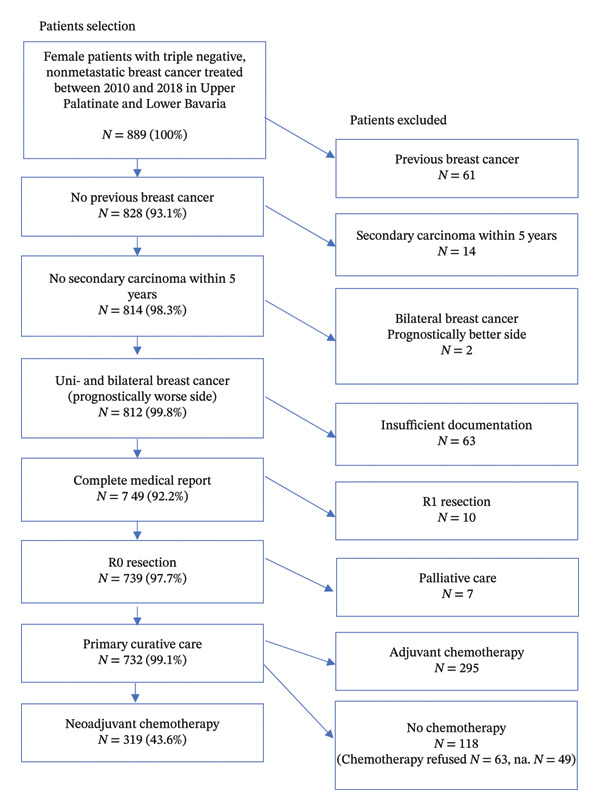
Depiction of the study collective.

Clinical cancer registry data from patients residing in the Upper Palatinate and Lower Bavaria regions, whose diagnoses of TNBC were registered by the Tumor Center Regensburg between January 1, 2010, and December 31, 2018, were analyzed. Figure 1 depicts inclusion and exclusion criteria for the final study collective. From the Tumor Center Regensburg, we obtained the following patient characteristics: Data on tumor stage, ER, PR, and Her2neu status, date of diagnosis of the primary tumor, age and age at diagnosis, menopausal status, last date of follow‐up, date of recurrence, date of death, Charlson comorbidity index, site location, lymphatic vessel invasion, vein invasion, histopathological cancer stage, grading, Ki67, treatment protocol for NACT, and completion of NACT or discontinuation of NACT. This dataset was also used to assess the outcome of patients with TNBC depending on the time intervals between diagnosis and the start of NACT [23], as well as between surgery and the initiation of adjuvant therapy [24].

### 2.2. Follow‐Up Definitions

Follow‐up for vital status and recurrence status was extended until December 31, 2023. Patient information, including demographic characteristics and variables related to cancer diagnosis and treatment, was abstracted from medical records by tumor registries as part of routine procedures.

*Neoadjuvant treatment regimens* Patients who received NACT were categorized into four different chemotherapy protocols: EC‐T, EC‐T/P, EC‐nabP/P, or other protocols, which included, for example, fluorouracil, epirubicin, and cyclophosphamide (FEC) and docetaxel, doxorubicin, and cyclophosphamide (DAC).

### 2.3. Primary and Secondary Endpoints

The primary endpoint was the rate of pCR, defined as the absence of invasive cancer in the breast and lymph nodes at surgery (ypT0/is ypN0). Secondary endpoints included recurrence‐free survival (RFS) and OS.

### 2.4. Statistical Methodology

The influence of the treatment regimen on the pCR rate was estimated using multivariable binary logistic regression, reported as odds ratios (ORs) with 95% confidence intervals (CIs). Covariates included age, comorbidities, tumor location, stage, grade, lymphovascular invasion, and Ki‐67 index. Descriptive statistics were used to evaluate the characteristics of the patient population according to treatment regimen subgroup. Follow‐up was calculated using the reverse Kaplan–Meier method. Survival time was calculated in years from the date of breast cancer diagnosis to the date of last follow‐up or the date of death. Patients who were alive at the study cutoff date of December 31, 2023, were censored on that date. The Tumor Center Regensburg regularly updates vital patient status information and active hospital follow‐up through linkages with state and national health offices and queries to the residents’ registration offices. RFS was determined as the difference between the date of diagnosis of the primary tumor and the last living date, recurrence date, date of death, or cutoff date. Univariable analyses of cumulative OS, cumulative recurrence rates, and RFS were conducted using the Kaplan–Meier method, and the log‐rank test was used for group comparisons. Cox proportional hazards regression was used for multivariable survival analysis. The 5‐year survival rates were analyzed, while median survival was not reached by the cutoff date. Missing data were handled via pairwise deletion, and no multiple imputation was performed because missingness was < 10% for key variables. Data collection and statistical analysis were performed using IBM SPSS Statistics Version 28.0 (IBM Corporation, Armonk, NY), with *p*‐values, and 95% CIs calculated for each model. All tests were two‐sided, and significance was set at *p* < 0.05.

### 2.5. Ethical Approval

This study was carried out in accordance with The Code of Ethics of the World Medical Association (Declaration of Helsinki). All members of the research team committed themselves to the confidentiality of the information provided as well as to data protection and are subject to medical confidentiality.

## 3. Results

### 3.1. Patients’ and Tumor Characteristics

A total of 732 patients with a diagnosis of Stages I–III TNBC between January 1, 2010, and December 31, 2018, were detected. The proportion of patients receiving NACT upon diagnosis of TNBC increased from 24% in 2010 to 73% in 2018. A total of 319 patients received NACT during the study period for early TNBC. The following analysis refers to these 319 patients who underwent NACT. Among them, 132 patients (41.4%) received NACT with EC‐T, 74 patients (23.2%) patients received NACT with EC‐P/T, and 22 patients (6.9%) received NACT with EC‐P/nabP. Other protocols of NACT were administered in 91 (28.5%) patients. The mean age at diagnosis was 53.5 years (median 53.4, interquartile range IQR 45.7–61.7 years). A total of 146 women (45.8%) were premenopausal, and 173 (54.2%) were postmenopausal at the time of diagnosis. A total of 16% had relevant comorbidities. Patient, tumor, and treatment characteristics stratified by administered NACT regimen are shown in Table 1.

**TABLE 1:** Patient, tumor, and treatment characteristics stratified by administered NACT regimen.

		Administered chemotherapy regimen									
		EC‐T		EC‐P/T		EC‐P/nabP		Others		Total	
		*N*	(%)	*N*	(%)	*N*	(%)	*N*	(%)	*N*	(%)
Age at diagnosis	< 40	17	12.9	18	24.3	8	36.4	8	8.8	51	16
	40–49	35	26.5	13	17.6	4	18.2	22	24.2	74	23.2
	50–59	46	34.8	25	33.8	6	27.3	27	29.7	104	32.6
	60–69	21	15.9	17	23.0	2	9.1	20	22.0	60	18.8
	70+	13	9.8	1	1.4	2	9.1	14	15.4	30	9.4

Menopausal status	Premenopausal	65	49.2	34	45.9	14	63.6	33	36.3	146	45.8
	Postmenopausal	67	50.8	40	54.1	8	36.4	58	63.7	173	54.2

Co‐morbidities	No	102	77.3	56	75.7	13	59.1	72	79.1	243	76.2
	Yes	16	12.1	13	17.6	8	36.4	14	15.4	51	16.0
	na	14	10.6	5	6.8	1	4.5	5	5.5	25	7.8

Stage	IA/B	25	18.9	21	28.4	10	45.5	13	14.3	69	21.6
	IIA	58	43.9	28	37.8	8	36.4	33	36.3	127	39.8
	IIB	29	22.0	15	20.3	3	13.6	25	27.5	72	22.6
	III	14	10.6	8	10.8	1	4.5	17	18.7	40	12.5
	na	6	4.5	2	2.7	0	0.0	3	3.3	11	3.4

Tumor size	T1	42	31.8	24	32.4	10	45.5	15	16.5	91	28.5
	T2	69	53.3	40	54.1	11	50.0	59	64.8	179	56.1
	T3	13	9.8	6	8.1	0	0.0	10	11.0	29	9.1
	T4	3	2.3	3	4.1	1	4.5	4	4.4	11	3.4
	TX/na	5	3.8	1	1.4	0	0.0	3	3.3	9	2.8

Nodal Status	N0	74	56.1	50	67.6	19	86.4	49	53.8	192	60.2
	N1	49	37.1	22	29.7	3	13.6	33	36.3	107	33.5
	N2	3	2.3	1	1.4	0	0.0	6	6.6	10	3.1
	N3	2	1.5	1	1.4	0	0.0	2	2.2	5	1.6
	na	4	3.0	0	0.0	0	0.0	1	1.1	5	1.6

Grading	G1/2	17	12.9	10	13.5	1	4.5	13	14.3	41	12.9
	G3	80	60.6	43	58.1	7	31.8	59	64.8	189	59.2
	GX/na	35	26.5	21	28.4	14	63.6	19	20.9	89	27.9

Ki67	0–25	28	21.2	12	16.2	9	40.9	20	22.0	69	21.6
	> 25	101	76.5	60	81.1	13	59.1	70	76.9	244	76.5
	na	3	2.3	2	2.7	0	0.0	1	1.1	6	1.9

pCR after NACT	No	75	56.8	33	44.6	5	22.7	47	51.6	160	50.2
	Yes	57	43.2	41	55.4	17	77.3	44	48.4	159	49.8

Type of surgery after NACT	BCT	117	88.6	61	82.4	15	68.2	75	82.4	268	84.0
	Mastectomy	14	10.6	12	16.2	7	31.8	16	17.6	49	15.4
	na	1	0.8	1	1.4	0	0.0	0	0.0	2	0.6
	Total	132	100	74	100	22	100	91	100	319	100

*Note:* EC‐T, epirubicin/cyclophosphamide followed by paclitaxel; EC‐P/T, epirubicin/cyclophosphamide followed by paclitaxel plus platinum; EC‐P/nabP, epirubicin/cyclophosphamide followed by nab‐paclitaxel plus platinum; NACT, neoadjuvant chemotherapy.

Abbreviations: na = not available, pCR = pathologic complete response.

### 3.2. Chemotherapy Protocol

**FIGURE 2:**
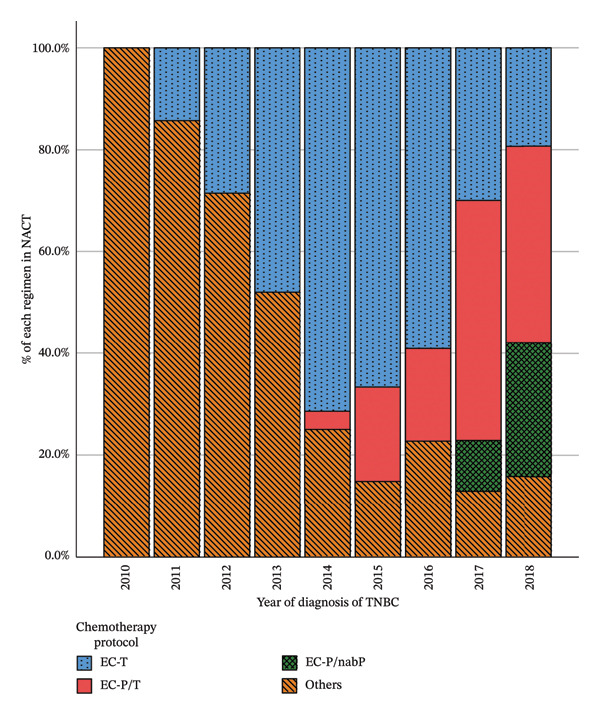
Applied NACT regimens in patients with early TNBC between 2010 and 2018.

Figure 2 depicts the evolution of administered chemotherapy regimens in patients with TNBC over the years. The first applications of platinum containing regimens took place in 2014. The proportion of platinum‐containing regimens increased from 3.6% in 2014 to 64.9% in 2018. The percentage of patients who received a combination of platinum with nabP instead of paclitaxel increased from 10% in 2017 to 26.3% in 2018.

Overall, 23.8% of patients discontinued NACT prematurely (see Table 2). Reasons for discontinuation included termination due to side effects, patients’ refusal, and disease progression. The highest number of discontinuations was in the subgroup of patients receiving EC‐P/nabP (59.1%); the administered chemotherapy regimen had a significant impact on the discontinuation rate (*p* ≤ 0.001).

**TABLE 2:** Discontinuation of therapy according to the administered NACT regimen.

Discontinuation of NACT	NACT regimen				
	EC‐T *N* (%)	EC‐P/T *N* (%)	EC‐P/nabP *N* (%)	Others *N* (%)	Total *N* (%)
Yes	19 (14.4)	21 (28.4)	13 (59.1)	23 (25.3)	76 (23.8)
No	110 (83.3)	52 (70.3)	9 (40.9)	67 (73.6)	238 (74.6)
na	3 (2.3)	1 (1.4)	0 (0.0)	1 (1.1)	5 (1.6)
Total	132 (100.0)	74 (100.0)	22 (100.0)	91 (100.0)	319 (100.0)

### 3.3. Pathologic Complete Response

Table 3 shows the results of the multivariable binary logistic regression for the likelihood of a pCR depending on the chemotherapy regimen (reference EC‐T, adjusted for age at diagnosis, comorbidities, tumor location, site localization, stage, grading, lymphatic and venous invasion, and Ki‐67 < 25 vs. ≥ 25%). A pCR occurred in 46.7% of the patients under NACT. In the subgroup without platinum, a pCR rate of 37.1% was achieved. Addition of platinum significantly increased the pCR rate to 54.1% compared to EC‐T (OR 3.476, 95% CI 1.655–7.300, *p* = 0.001). EC‐P/nabP led to a significant increase in the pCR rate to 77.3% (OR 8.767, 95% CI 2.421–31.744, *p* ≤ 0.001).

**TABLE 3:** pCR rates and odds ratios from multivariable binary logistic regression for the likelihood of a pCR depending on the chemotherapy regimen.

NACT regimen	Non‐pCR *N* (%)	pCR *N* (%)	Total N	p‐value	Odds ratio	95% CI
EC‐T	83 (62.9)	49 (37.1)	132		1.000	
EC‐P/T	34 (45.9)	40 (54.1)	74	< 0.001	3.476	1.655–7.300
EC‐P/nabP	5 (22.7)	17 (77.3)	22	< 0.001	8.767	2.421–31.744
Others	48 (52.7)	43 (47.3)	91	0.055	1.989	0.985–4.016
Total	170 (53.3)	149 (46.7)	319			

A subgroup analysis comparing the use of nabP versus paclitaxel, each in combination with carboplatin, in a multivariable binary logistic regression for the likelihood of a pCR (EC‐P/nabP vs. EC‐T/P, adjusted for age at diagnosis, comorbidities, tumor location, site localization, stage, grading, lymphatic and venous invasion, and Ki‐67 < 25 vs. ≥ 25%) yielded no significant difference in reaching pCR (OR 2.354, 95% CI 0.454–12.188). In a multivariable subgroup analysis restricted to 238 patients with regularly completed chemotherapy, all regimens revealed a significantly higher rate of pCR compared to EC‐T (Table A1).

### 3.4. Survival According to Pathologic Response

The median follow‐up was 4.8 years (mean 4.9). The estimated 5‐year OS in the complete cohort of 319 patients was 74.5%; the 5‐year RFS rate was 66.5%. Attaining a pCR significantly influenced OS (5‐year rate 81.0% in patients with pCR vs. 69.1% in patients with no pCR; *p* = 0.018, Figure 3(a)). It provided a highly significant benefit for RFS (5‐year rate 79.7% vs. 56.2%; *p* < 0.001, Figure 3(b)). The Kaplan–Meier results could not be confirmed in multivariable Cox regression analyses: After adjusting for confounders, the HR for OS in patients with pCR was 1.176 (95% CI 0.623–2.221; *p* = 0.617) compared to patients not attaining pCR. The corresponding HR for RFS was 0.786 (95% CI 0.450–1.372; *p* = 0.398).

FIGURE 3Kaplan–Meier survival estimates: (a) OS estimates according to pCR. (b) RFS estimates according to pCR. (c) OS estimates according to chemotherapy regimen. (d) RFS estimates according to chemotherapy regimen.

**Figure:**
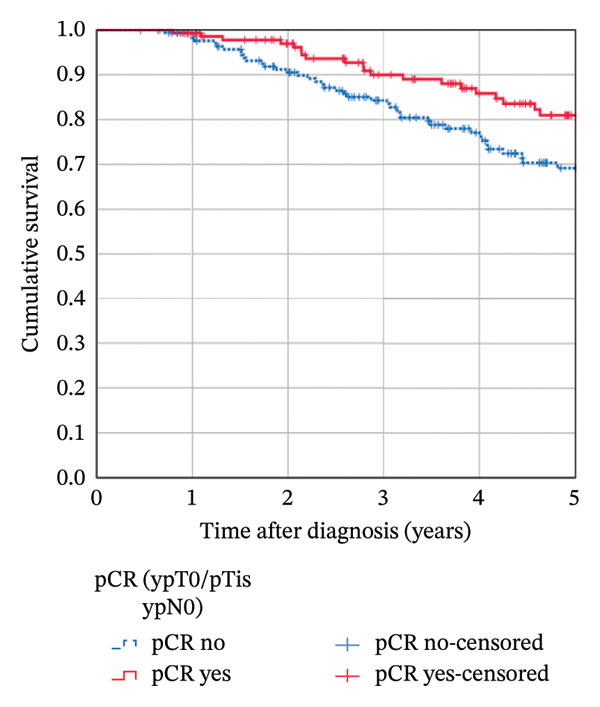
(a)

**Figure:**
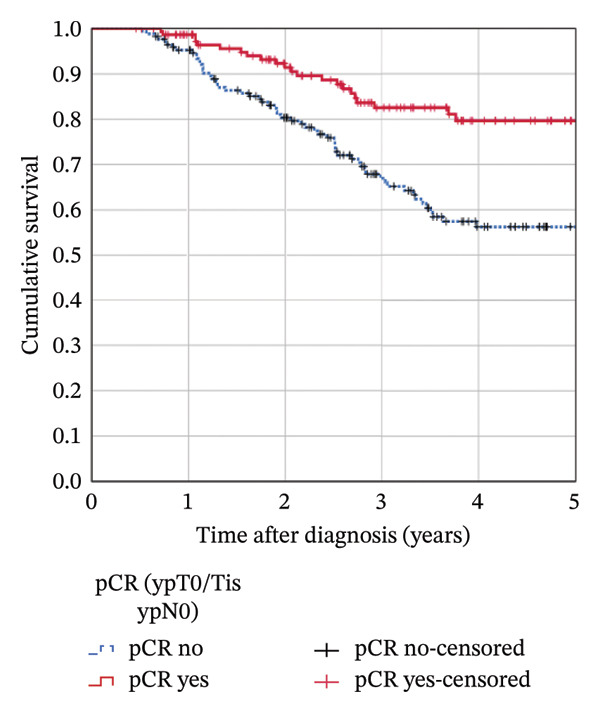
(b)

**Figure:**
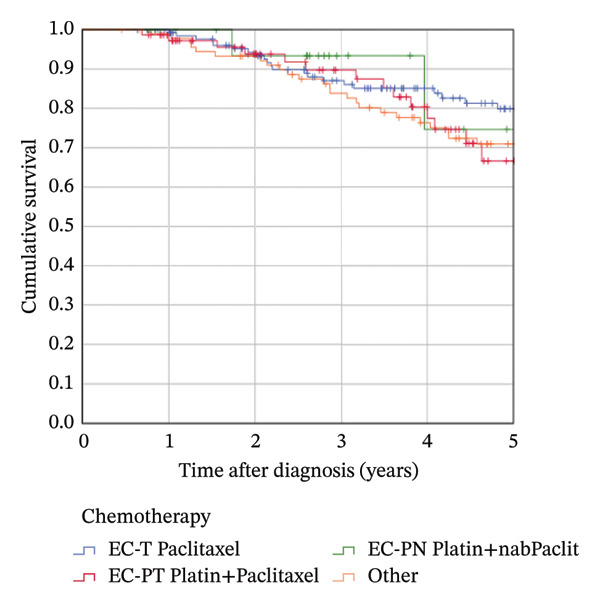
(c)

**Figure:**
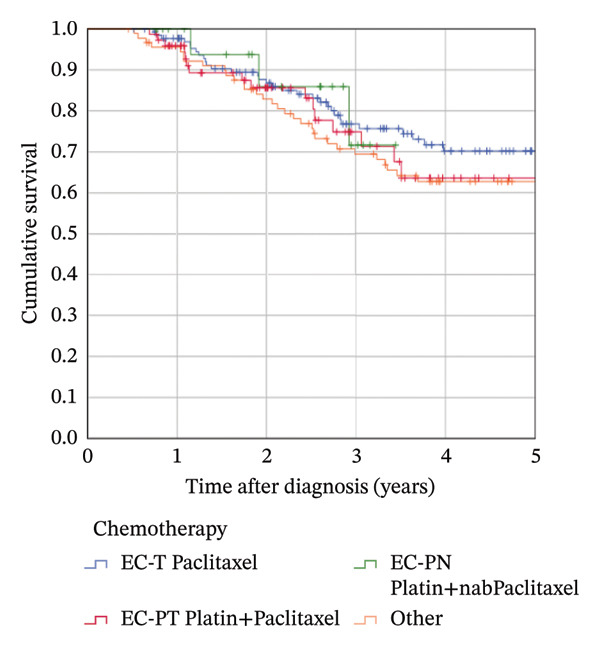
(d)

### 3.5. Survival According to Chemotherapy Regimen

The estimated 5‐year OS was 79.9% in the EC‐T group, 66.6% in the EC‐PT group, and 74.7% in the EC‐PnabP group. The use of platinum did not have a significant impact on OS. No significant differences were observed in Kaplan–Meier analysis, neither in univariable nor in multivariable Cox regression analyses (Figure 3(c), Table A2). The subgroup analysis with 238 patients receiving complete chemotherapy revealed no significant differences as well. The estimated 5‐year RFS was 70.2% in the EC‐T group, as compared with 63.6% in the EC‐P/T group and 71.6% in the EC‐P/nabP group. Patients receiving other regimens had an estimated 5‐year RFS of 62.8%. The administered NACT regimen showed no significant differences in reached RFS rates (Figure 3(d), Table A3), confirmed by multivariable Cox regression analysis and subgroup analysis in 238 patients receiving complete chemotherapy.

## 4. Discussion

In the present study we analyzed real‐world data from a large cancer registry to describe the clinical practice regarding NACT regimens and their resulting outcomes in patients with early TNBC. Patients were treated between 2010 and 2018. Overall, 46.7% of the patients under NACT reached a pCR. This is higher than in the largest study to date on use and effects of NACT in German breast cancer centers, which was recently published by Ortmann et al. [1]. They analyzed data from 94,638 patients treated at 55 breast cancer centers to describe the current clinical practice and outcomes after NACT under routine conditions and found a pCR rate of 38% in patients with TNBC, which is similar to our platinum‐free cohort, where pCR rates of 37.1% were reached. However, this large multicenter analysis did not include an analysis of the specific substances used, which is addressed in the present study. We could demonstrate that the use of platinum in the neoadjuvant setting for early TNBC increased significantly over time and progressively rose over the years, with a proportion of 3.6% in 2014 reaching levels up to 64.9% in 2018. The achievement of pCR was used as a surrogate marker in our study. The addition of platinum led to an increase in pCR rates to 54.1%. Achieving a pCR was associated with a longer 5‐year OS (81.0%) compared with patients who did not achieve a pCR (69.1%, *p* = 0.018). Referring to RFS, the difference was even more pronounced with a 5‐year rate of 79.7% vs. 56.2% (*p* < 0.001). The Kaplan–Meier results could not be confirmed in multivariable Cox regression analyses: After adjusting for confounders, no significant difference regarding OS and RFS was observed between patients with or without pCR. The covariates included age, comorbidities, tumor location, stage, grade, lymphovascular invasion, and Ki‐67 index. The follow‐up ended at the end of 2023, so the shortest follow‐up was 5 years. Follow‐up was conducted until the end of 2023. Thus, depending on the date of diagnosis, patients were followed for a period ranging from 5 to 13 years. It is possible that with a longer follow‐up, survival according to pCR might also reach statistical significance in the multivariable analysis. However, it must be noted here that we did not collect data on supplementary post‐NACT, which could impact EFS and long‐term outcomes. As the CREATE‐X trial data on postneoadjuvant therapy emerged in 2017 [6] and our study ended in 2018, its effect seems negligible. Regarding the pCR rates, there is a high concordance with published study data. The pCR rates, achieved in the GeparSixto study by the addition of platinum, were 53%, translating into an absolute benefit in 3‐year EFS of 9.7% compared with the platinum‐free control arm [14]. The results of the GeparSixto study were first published in 2014, the same year when patients in our study collective first received platinum‐containing NACT for TNBC. Published in 2015, the CALGB/Alliance 40,603 study raised the pCR rate to 54% by adding carboplatin [13]. In the BrighTNess trial, which was first published in 2018 [15], the pCR rate in the veliparib and carboplatin arm was 53%, in the carboplatin arm 58%, and 31% in the standard of care. All these findings support the addition of carboplatin to weekly paclitaxel followed by AC NACT for early‐stage TNBC. We were able to demonstrate that this regimen was increasingly adopted into clinical practice shortly after the publications of the respective studies. As early as 2017, 47.1% of patients of our study collective with early TNBC received NACT with this exact regimen, with an additional 10% receiving a similar regimen but with nabP instead of paclitaxel. The introduction of nabP [21] led to increased adoption in some centers. In our study, its combination with platinum achieved a 77.3% pCR rate, compared to 54.1% receiving platinum with solvent‐based paclitaxel. While the nabP subgroup was small (*n* = 22), it indicates clear benefits. NabP’s advantages, a solvent‐free, albumin‐stabilized paclitaxel, include enhanced drug concentrations in tumors [25], improved tissue penetration [26], slower elimination, and potential immunostimulation [27]. Both the biological rationale and the data from our study support the findings of the GeparSepto trial [21]. Although the small subgroup of only 22 patients receiving the EC‐P/nab‐paclitaxel regimen in our study achieved a very high pCR rate of 77.3%, these results can only be considered exploratory due to the small sample size and must be interpreted with caution. However, these insights into the use of nabP instead of paclitaxel diminish with the results of the Keynote 522 study, where solvent‐based paclitaxel was utilized [22]. This trial investigated pembrolizumab’s addition to NACT for Stages II–III TNBC, achieving a pCR of 64.8% (vs. 51.2% with placebo) among 602 patients. This marks the highest pCR rates achieved in a Phase 3 study for high‐risk TNBC. Patients achieving pCR experienced significantly improved EFS [28]. Likewise, the IMpassion 031 study showed promising results with atezolizumab [29]. These findings advocate for immunotherapy in Stages II–III TNBC treatment, with protocols aligned with the Keynote 522 trial being recommended in national and international guidelines [20, 30]. In addition to the various chemotherapy regimens in the neoadjuvant setting, evidence for postneoadjuvant therapy has also increased in recent years. The CREATE‐X study demonstrated the benefit of capecitabine in patients who did not achieve a pCR through NACT [6], and the OlympiA study confirmed the benefit of Olaparib in patients with pathogenic BRCA1 or BRCA2 mutations and no pCR after NACT [5].

Depending on the chemotherapy regimen administered, relatively high rates of treatment discontinuation were observed. The highest discontinuation rate, at 59%, was recorded in the EC‐P/nabP group. However, it should be noted that this subgroup comprised only 22 patients, which somewhat limits the interpretability of these findings. We did not analyze the exact timing of premature discontinuation of NACT. Based on clinical experience, poor tolerability of platinum agents or taxanes combined with a good response to NACT often leads to the omission of the final one or two chemotherapy cycles in order to mitigate toxicity, although in such cases—compared with earlier discontinuation—a high mean relative total dose intensity (mRTDI) is still achieved. In the GeparSixto trial [5], which investigated the EC‐P/nabP regimen, relatively high discontinuation rates were also reported among patients with TNBC, with 49% in the platinum‐containing arm compared with 36% in the platinum‐free arm. Moreover, a higher incidence of both hematological and nonhematological adverse events was observed in the carboplatin arm than in the noncarboplatin arm, including neutropenia, anemia, and thrombocytopenia, as well as diarrhea and nausea.

As depicted, oncological treatments are becoming increasingly complex with new studies expanding therapeutic options. Regular reassessment of how study data are integrated into clinical practice, as done in this study, is crucial and leads to gaining new insights into how quickly and completely new study data are being implemented in everyday clinical settings. If it becomes apparent that certain findings are not optimally implemented, optimizations for the future can be derived from this. For instance, managing side effects in real‐world settings may differ from studies, causing higher therapy discontinuation. Since real‐world data of this nature are rarely analyzed, the presented data hold particular value. Firstly, it is worth mentioning the large number of patients with TNBC for whom fully evaluable data on the type of neoadjuvant therapy were available. Secondly, the study period from 2010 to 2018 coincides with a time frame in which many developments in the field of neoadjuvant therapy for TNBC occurred, as outlined above. This provides insight into how quickly new study findings are translated into clinical practice. A further benefit of the presented data sourced from the mandatory clinical cancer registry is that it documents data on patients treated in certified breast cancer units and data from patients treated outside certified centers. Thus, the reality appears to be depicted without distortion. The presented real‐world findings provide a pre‐immunotherapy benchmark for early‐stage TNBC, reflecting how clinical trial innovations, namely the integration of platinum agents and nab‐paclitaxel, were translated into routine practice prior to the adoption of immune checkpoint inhibitors. By documenting treatment patterns, pCR rates, and survival outcomes in this context, our study establishes a reference point against which postpembrolizumab outcomes can be compared. Such comparisons may help to contextualize the incremental benefits of immunotherapy in everyday clinical settings, identify patient subgroups who derive the most advantage, and guide optimization of neoadjuvant and postneoadjuvant strategies in contemporary practice. The current study has several limitations that must be acknowledged. First, this was a retrospective, single‐center analysis using registry data, which limits causal inference and generalizability beyond the covered geographic region. It must be considered that the present data pertain to two regions in Germany; to draw conclusions about nationwide healthcare provision, multiple cancer registries would need to be jointly analyzed. Additionally, it should be noted that the data collected from registries never possess the same quality as in clinical trials, which must be considered during interpretation. Second, toxicity and adverse event data were not captured in sufficient detail to evaluate regimen tolerability. Since toxicity strongly influences treatment completion, this may bias comparative analyses, particularly in the EC‐P/nabP subgroup with higher discontinuation rates. Third, postneoadjuvant treatments (e.g., capecitabine or olaparib) were incompletely recorded due to the cutoff date preceding widespread adoption of these agents. This limits assessment of long‐term survival benefit and may contribute to the attenuated effects observed in multivariable survival models. Fourth, critical variables such as BRCA status and other molecular characterizations, as well as socioeconomic and demographic factors, were incomplete or missing. These omissions may influence both pCR and survival interpretations and therefore warrant caution when interpreting the results. Despite these limitations, the registry‐based design provides a robust reflection of real‐world practice and complements evidence from clinical trials by illustrating how study results are implemented at the population level. Considering the current situation, in which immune checkpoint inhibitors have firmly established their role in the treatment of TNBC, the presented data provide a historical perspective. The analysis of real‐world data since the introduction of immune checkpoint inhibitors is underway and will provide valuable insights into current practice as well as the acceptance of the therapy outside of clinical trials.

## 5. Conclusion

This is to the best of our knowledge the first real‐world study to examine the outcome differences between different NACT regimens applied in patients with early TNBC in the 2010s of the 21^st^ century. A successful translation of study findings into clinical practice has been demonstrated. The use of platinum salts was effectively integrated into clinical practice. As treatment for TNBC becomes increasingly complex, clinical cancer registry data remain invaluable for assessing and potentially improving patient care in the future.

## 6. Clinical Practice Points

• Study findings were swiftly incorporated into routine clinical practice, leading to improved patient outcomes.
• Particularly the use of platinum in the neoadjuvant setting for early‐stage TNBC has significantly increased over time, alongside a rising pCR rate.
• Oncological treatments are becoming increasingly complex, highlighting the importance of regularly reassessing how study data are integrated into clinical practice.

## Funding

No funding was received for this manuscript. Open access funding was enabled and organized by Projekt DEAL.

## Ethics Statement

This study was carried out in accordance with the Code of Ethics of the World Medical Association (Declaration of Helsinki). All members of the research team committed themselves to the confidentiality of the information provided as well as to data protection and are subject to medical confidentiality.

## Conflicts of Interest

Maria Eleni Hatzipanagiotou has received honoraria for lectures and/or consulting from Novartis, Lilly, Roche, Pfizer, and AstraZeneca. Olaf Ortmann is on the board of the German Cancer Society. He received speaker honoraria from MSD SHARP & DOHME GMBH, Verband Forschender Arzneimittelhersteller (vfa), Novo Nordisk, AstraZeneca, Aurikamed, Med Update, RG Ärztefortbildung, and Pierre Fabre Pharma GmbH and holds stock in Bayer, Novartis, CureVac, and Fresenius. Stephan Seitz has received speaker honoraria from AstraZeneca, GE, GSK, IGEA, Lilly, MSD, Novartis, Pfizer, and Roche and honoraria for consulting from AstraZeneca, GSK, Lilly, MSD, Novartis, Pfizer, and Roche. The other authors declare no conflicts of interest.

## Supporting Information

Table A1 presents the pCR rates and the corresponding odds ratios from the multivariable binary logistic regression analysis, evaluating the likelihood of achieving pCR based on the NACT regimen in patients who completed chemotherapy as scheduled. Table A2 displays OS outcomes stratified by the administered NACT regimen, derived from the multivariable Cox regression analysis.

Table A3 presents RFS outcomes according to the administered NACT regimen, also based on multivariable Cox regression analysis.

## Supporting information


**Supporting Information** Additional supporting information can be found online in the Supporting Information section.

## Data Availability

The data that support the findings of this study are available on request from the corresponding author. The data are not publicly available due to privacy or ethical restrictions.
