# Supplementary material for: Treatment Regimens and Response Rates in Early TNBC: A Review of Real‐World Practice in the Second Decade of the 21st Century
Source: Breast J. 2026 Apr 8;2026:9970072. doi: 10.1155/tbj/9970072 (PMC13059670; doi:10.1155/tbj/9970072)
Supplement: Supplementary file 1 — Supporting Information Additional supporting information can be found online in the Supporting Information section. [file TBJ-2026-9970072-s001.docx]

Appendices

| **NACT regimen** | **pCR not reached**  **N (%)** | **pCR reached**  **N (%)** | **Total** | ***p*-value** | **Odds Ratio** | **95% CI** |
| --- | --- | --- | --- | --- | --- | --- |
| **EC – T** | 66 (60.0) | 44 (40.0) | 110 |  | 1.000 |  |
| **EC – P/T** | 22 (42.3) | 30 (57.7) | 52 | 0.001 | 4.602 | 1.819-11.644 |
| **EC – P/nabP** | 2 (22.2) | 7 (77.8) | 9 | 0.003 | 16.793 | 2.617-107.763 |
| **Others** | 29 (43.3) | 38 (56.7) | 67 | 0.008 | 3.057 | 1.343-6.956 |
| **Total** | 119 (50.0) | 119 (50.0) | 238 |  |  |  |

Table A1 pCR rates and odds ratios from multivariable binary logistic regression for the likelihood of a pCR depending on the chemotherapy regime in patients with regularly completed chemotherapy

| **NACT regimen** | **Total**  **N** | **Event**  **N** | ***p*-value** | **Hazard Ratio** | **95% CI** |
| --- | --- | --- | --- | --- | --- |
| **EC – T** | 132 | 26 |  | 1.000 |  |
| **EC – P/T** | 74 | 14 | 0.122 | 1.795 | 0.856-3.767 |
| **EC – P/nabP** | 22 | 2 | 0.451 | 1.806 | 0.388-8.409 |
| **Others** | 91 | 30 | 0.292 | 1.364 | 0.766-2.429 |
| **Total** | 319 | 72 |  |  |  |

Table A2 OS according to the administered NACT regimen – result from multivariable Cox-regression (adjusted for age at diagnosis, comorbidities, tumor location, site localization, stage, grading, lymphatic and venous invasion, Ki-67 <25 vs >= 25%

| **NACT regimen** | **Total**  **N** | **Event**  **N** | ***p*-value** | **Hazard Ratio** | **95% CI** |
| --- | --- | --- | --- | --- | --- |
| **EC – T** | 132 | 35 |  | 1.000 |  |
| **EC – P/T** | 74 | 16 | 0.289 | 1.424 | 0.741-2.737 |
| **EC – P/nabP** | 22 | 3 | 0.362 | 1.818 | 0.504-6.562 |
| **Others** | 91 | 37 | 0.490 | 1.201 | 0.714-2.020 |
| **Total** | 319 | 91 |  |  |  |

Table A3 RFS according to the administered NACT regimen – result from multivariable Cox-regression (adjusted for age at diagnosis, comorbidities, tumor location, site localization, stage, grading, lymphatic and venous invasion, Ki-67 <25 vs >= 25%
